# The Ability of AST-120 to Lower the Serum Indoxyl Sulfate Level Improves Renal Outcomes and the Lipid Profile in Diabetic and Nondiabetic Animal Models of Chronic Kidney Disease: A Meta-Analysis

**DOI:** 10.3390/toxins16120544

**Published:** 2024-12-16

**Authors:** Hande O. Altunkaynak, Eda Karaismailoglu, Ziad A. Massy

**Affiliations:** 1 Department of Pharmacology, Gulhane Faculty of Pharmacy, University of Health Sciences, 06018 Ankara, Turkey; 2 Department of Medical Informatics, Gulhane Faculty of Medicine, University of Health Sciences, 06018 Ankara, Turkey; eda.karaismailoglu@sbu.edu.tr; 3 Association pour L’utilisation du rein Artificiel en Région Parisienne (AURA), 75014 Paris, France; ziad.massy@auraparis.org; 4 Department of Nephrology, CHU Ambroise Paré, APHP, Boulogne Billancourt, 92100 Paris Cedex, France; 5 Centre for Research in Epidemiology and Population Health, University Paris-Saclay, University Versailles-Saint Quentin, Inserm UMRS 1018, Clinical Epidemiology Team, 94000 Villejuif, France

**Keywords:** AST-120, indoxyl sulfate, chronic kidney disease, diabetes, renal function markers, cholesterol, triglyceride, rat, mouse, meta-analysis

## Abstract

The therapeutic benefit of the oral adsorbent drug AST-120 in chronic kidney disease (CKD) is related to an indoxyl sulfate (IS)-lowering action. Diabetes and dyslipidemia might worsen kidney damage in CKD. However, it is not known whether AST-120 influences lipid abnormalities as well as renal function in patients with CKD and diabetes. The objective of the present meta-analysis was to evaluate the efficacy of AST-120 treatment in CKD using data from preclinical studies. Mixed-effect or random-effect models were used to estimate the standardized mean difference (SMD) and the 95% confidence interval (CI). Publication bias was assessed with a funnel plot and Egger’s test. The potential influence of some variables (the dose and duration of AST-120 treatment, the animal species, and the CKD model’s diabetic status) was evaluated in subgroup analyses. Treatment with AST-120 was associated with a significantly lower IS level in animals with CKD (SMD = −1.75; 95% CI = −2.00, −1.49; *p* < 0.001). Significant improvements in markers of renal function and the lipid profile were also observed. In subgroup analyses of the cholesterol level, the diabetic status, the AST-120 dose, and the animal species were found to be influential factors. AST-120 lowered serum IS and triglyceride levels and improved renal function in animal models of CKD independent of diabetes status. However, AST-120’s ability to lower the total cholesterol level was more prominent in animals with diabetic CKD.

## 1. Introduction

AST-120 is an orally administered adsorbent drug with beneficial effects on renal function in people with chronic kidney disease (CKD) [1]. The therapeutic benefit of AST-120 is linked to its ability to counter the accumulation of various gut-derived uremic toxins (such as indoxyl sulfate (IS) and p-cresyl sulfate (PCS)) in the body. AST-120 binds to indole, prevents its absorption (thereby inhibiting its conversion to IS in the liver), and facilitates its excretion in the feces [1,2,3]. IS is one of the most extensively studied uremic toxins in view of its detrimental effects on both the kidneys and the vasculature in CKD [3]. IS contributes to renal and/or vascular dysfunction through a number of pathways, including inflammation, fibrosis, and oxidative stress [2,3]. Since IS is primarily eliminated by the kidneys, it accumulates in parallel with the decline in renal function in CKD. A growing body of evidence indicates that elevated blood levels of IS are associated with worse renal and cardiovascular outcomes in CKD [3,4,5]. Moreover, an elevated IS level was found to be associated with a greater risk of all-cause mortality in patients with CKD [4]. In this regard, the results of preclinical and clinical studies have shown that AST-120 is effective in lowering blood IS levels in CKD [1,6]. It has also been indicated that AST-120 can delay the progression of CKD, as reflected by a slower decrease in the estimated glomerular filtration rate and a reduction in serum creatinine levels [1]. Furthermore, it has been suggested that AST-120 can delay the progression of glomerular sclerosis and interstitial fibrosis [5]. Taken as a whole, the current evidence suggests that AST-120 has beneficial effects on renal function, as indicated by common markers of CKD progression: the creatinine clearance rate, the serum creatinine level, and blood urea nitrogen (BUN) and urine protein levels [1,2].

Dyslipidemia is frequently observed in patients with CKD and might accelerate the progression of renal functional impairment and cardiovascular disease [7]. A previously published study indicated that certain uremic toxins were associated with an abnormal lipid profile in patients with kidney failure; IS was inversely associated with total, non-HDL, HDL, and LDL cholesterol levels [8]. Along with dyslipidemia, hypertension is known to be a crucial factor in the onset and progression of CKD [7]. However, it has not been determined whether uremic toxins influence the pathological structural alterations in glomeruli (rather than in the renal vasculature) in CKD. It has been suggested that AST-120 can work concomitantly with antihypertensive medications (such as angiotensin-converting enzyme inhibitors) to slow CKD progression [1]. However, it is still not clear whether high blood pressure has a negative influence on the beneficial renal effects of AST-120 as CKD progresses.

The results of prospective clinical trials and retrospective observational studies are inconsistent with regard to the renoprotective effects of AST-120 in patients with CKD [5]. Furthermore, little is known about the efficacy of AST-120 in diabetic patients with CKD or whether AST-120 has any influence on the lipid profile. When evaluating the efficacy of AST-120 in clinical trials, it is difficult to standardize a number of parameters; these include the cause and severity of CKD and the types of medication being taken concomitantly. We reasoned that preclinical studies of this topic might be useful for excluding factors that interfere with AST-120 treatment and thus accurately assessing the drug’s efficacy in appropriate animal models of CKD.

The objective of the present study was therefore to evaluate the effects of AST-120 treatment on renal outcomes and the lipid profile. To this end, we performed a meta-analysis of data from studies of diabetic and nondiabetic animals with CKD.

## 2. Results

**Figure 1:**
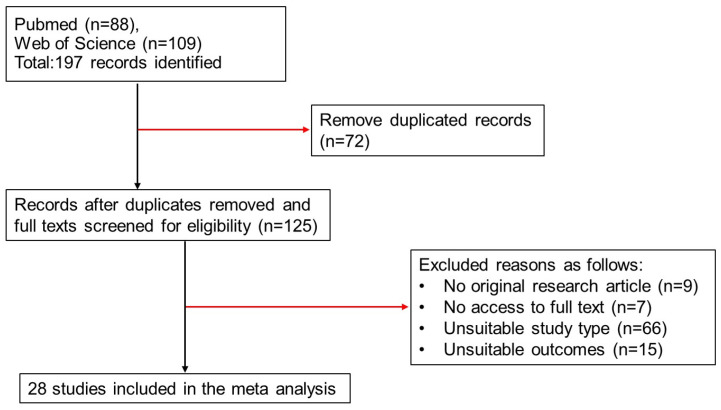
A flow chart of the literature search and study selection.

The results of this meta-analysis are reported in line with the Preferred Reporting Items for Systematic Review and Meta-Analyses guidelines [9] (Table S1). The search strategy defined in detail in Section 5 yielded a total of 197 records (Figure 1). After the removal of duplicates, 97 of the remaining 125 articles met one or more exclusion criteria and were excluded. We did not have access to the full text of some potentially includable publications. Hence, data from 28 publications were included in the meta-analysis [6,10,11,12,13,14,15,16,17,18,19,20,21,22,23,24,25,26,27,28,29,30,31,32,33,34,35,36].

### 2.1. The Characteristics of the Studies Included in the Meta-Analysis

Data from 545 animals (rats or mice) in the 28 studies published between 2000 and 2023 were selected (Table 1). The animals used were Sprague–Dawley rats, Wistar rats, Otsuka Long-Evans Tokushima Fatty (OLETF) rats, C57BL/6J mice, and ApoE−/− mice. Both males and females were studied, and the number of animals ranged from 4 to 17 per group. Subtotal nephrectomy was the most common means of inducing CKD. The AST-120 was administered orally (at doses of 1 g/day, 4 g/kg, or 4.5 g/kg), or animal chow containing AST-120 (at doses ranging from 4% *w*/*w* to 8% *w*/*w*). The duration of the AST-120 treatment and follow-up ranged from 3 days to 65 weeks.

### 2.2. The Effect of AST-120 on the Serum IS Level

**Figure 2:**
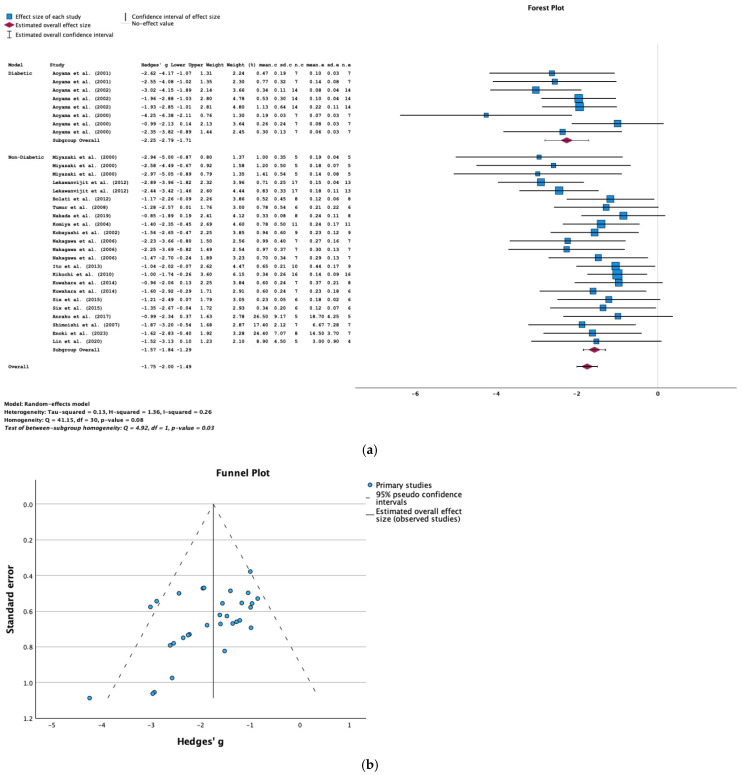
(**a**) A forest plot of data from studies of the effect of AST-120 on the serum IS levels in subgroups of animals (diabetic vs. nondiabetic animals with CKD) (sd: standart deviation; n: number; c: comparator (CKD group); e: experimental (CKD-AST-120 group)). (**b**) A funnel plot of data from studies of the effect of AST-120 on the serum IS levels in animals with CKD.

The data used to evaluate the effect of AST-120 on the serum IS level came from 19 studies. According to the result of the meta-analysis, AST-120 was associated with a significantly lower serum IS level in animals with CKD (SMD = −1.75; 95% CI = −2.00, −1.49; *p* < 0.001; I^2^ = 26%) (Figure 2a).

We performed sub-analyses for four variables: the AST-120 dose (4 g/kg/BW, animal chow containing 4–5% *w*/*w* or 8% *w*/*w* AST-120), the animal species (rat vs. mouse), the diabetic status of the animal model (diabetic CKD vs. nondiabetic CKD), and the duration of the AST-120 treatment. With regard to the animal model, a significantly lower IS level was found in the treated diabetic and nondiabetic animals with CKD (SMD = −2.25, *p* < 0.001; SMD = −1.57, *p* < 0.001, respectively, Figure 2a). However, there was no significant difference for the other three variables, i.e., the AST-120 dose (4 g/kg/BW, animal chow containing 4–5% *w*/*w* or 8% *w*/*w* AST-120), the duration of treatment, and the animal species (rat vs. mouse) (*p* > 0.05).

A possible statistically significant impact of publication bias on the variables was determined using Egger’s test and a funnel plot. The results show that there was no significant publication bias in the studies examined (Egger’s test; *p* = 0.558) (Figure 2b).

### 2.3. The Effect of AST-120 on Renal Function Markers

#### 2.3.1. The Serum Creatinine Level

**Figure 3:**
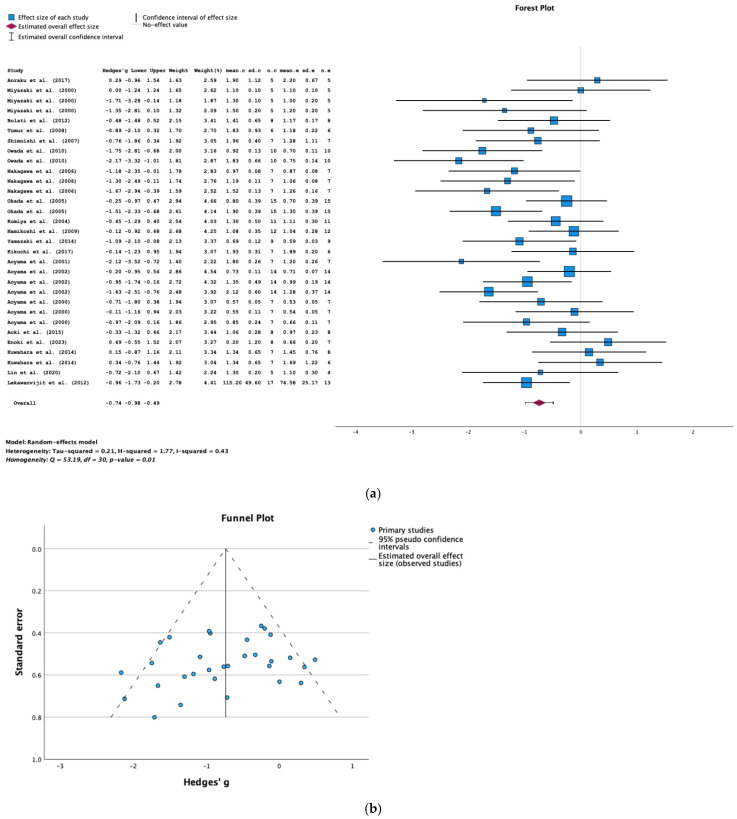
(**a**) A forest plot of data from studies of the effect of AST-120 on the serum creatinine levels in animals with CKD (sd: standart deviation; n: number; c: comparator (CKD group); e: experimental (CKD-AST-120 group)). (**b**) A funnel plot for studies of the effect of AST-120 on the serum creatinine levels in animals with CKD.

In a meta-analysis of data from 20 studies, AST-120 was associated with a significantly lower serum creatinine level in animals with CKD (SMD = −0.74; 95% CI = −0.98, −0.49; *p* < 0.001; I^2^ = 43%) (Figure 3a). The results of Egger’s test for possible publication bias were not significant (*p* = 0.834) (Figure 3b).

#### 2.3.2. The Creatinine Clearance Rate

**Figure 4:**
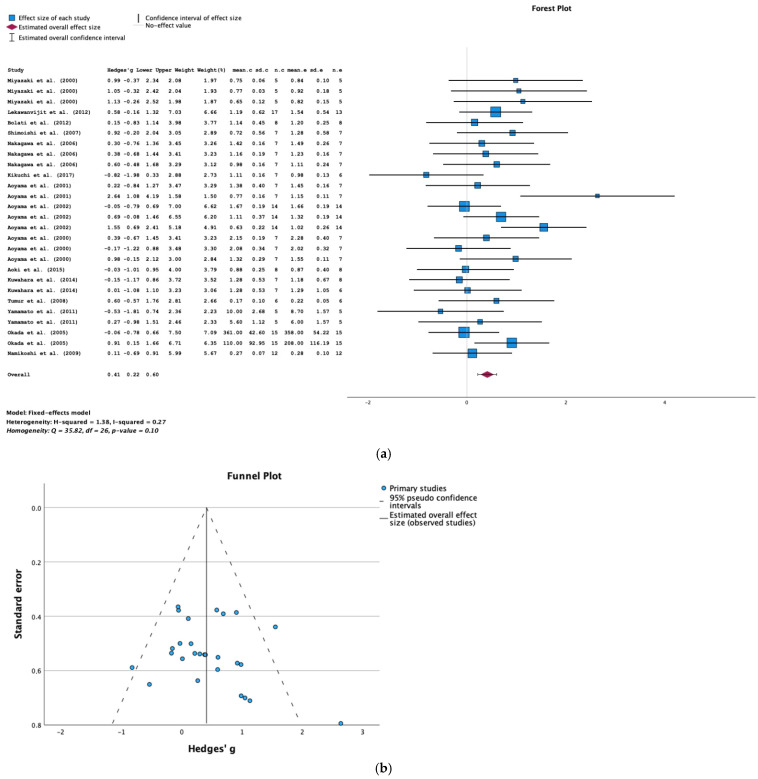
(**a**) A forest plot of data from studies of the effect of AST-120 on creatinine clearance in animals with CKD (sd: standart deviation; n: number; c: comparator (CKD group); e: experimental (CKD-AST-120 group)). (**b**) A funnel plot for studies of the effect of AST-120 on creatinine clearance in animals with CKD.

The result of the meta-analysis of data from 15 studies demonstrated that AST-120 treatment was associated with significantly greater creatinine clearance in animals with CKD (SMD = 0.41; 95% CI = 0.22, 0.60; *p* < 0.001; I^2^ = 27%) (Figure 4a).

The funnel plot and Egger’s test (*p* = 0.868) did not show evidence of publication bias in the studies of creatinine clearance (Figure 4b).

#### 2.3.3. BUN

**Figure 5:**
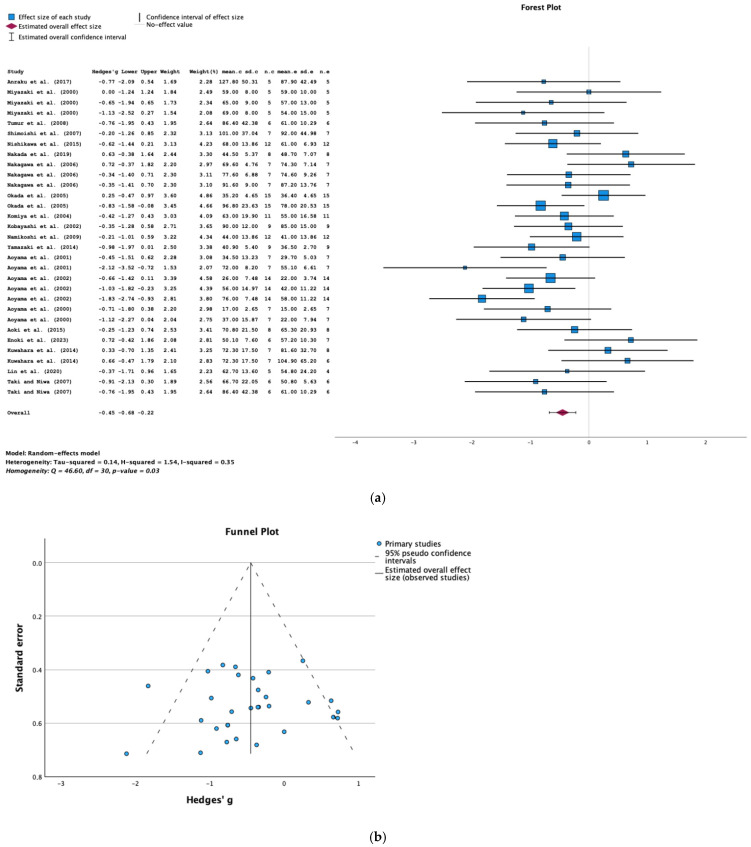

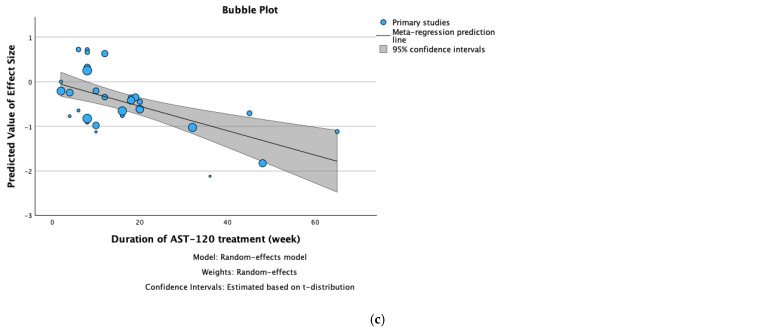
(**a**) A forest plot of data from studies of the effect of AST-120 on the BUN levels in animals with CKD (sd: standart deviation; n: number; c: comparator (CKD group); e: experimental (CKD-AST-120 group)). (**b**) A funnel plot of data from studies of the effect of AST-120 on the BUN levels in animals with CKD. (**c**) A bubble plot of data from studies of the effect of the duration of AST-120 treatment on the BUN levels in animals with CKD.

The results of the meta-analysis of data from 20 studies show that AST-120 treatment was associated with a significantly lower BUN level (SMD = −0.45; 95% CI = −0.68, −0.22; *p* < 0.001; I^2^ = 35%) (Figure 5a). The funnel plot and Egger’s test (*p* = 0.774) did not show evidence of publication bias in the studies of BUN levels (Figure 5b).

Furthermore, a subgroup analysis with regard to the duration of AST-120 treatment indicated that a longer duration was associated with greater decreases in BUN levels (Figure 5c).

#### 2.3.4. Proteinuria

**Figure 6:**
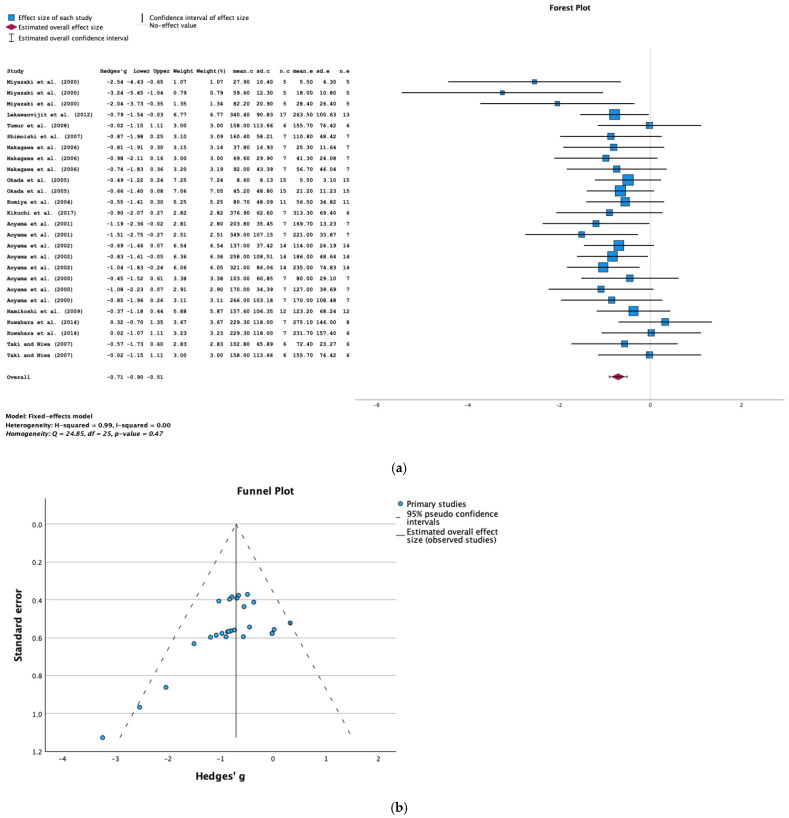
(**a**) A forest plot of data from studies of the effect of AST-120 on proteinuria in animals with CKD (sd: standart deviation; n: number; c: comparator (CKD group); e: experimental (CKD-AST-120 group)). (**b**) A funnel plot of studies of the effect of AST-120 on proteinuria in animals with CKD.

The results of a meta-analysis of data from 14 studies show that AST-120 was associated with significantly lower proteinuria in animals with CKD (SMD = −0.71; 95% CI = −0.90, −0.51; *p* < 0.001; I^2^ = 0%) (Figure 6a).

A funnel plot and Egger’s test (*p* = 0.535) did not show evidence of publication bias in the studies of proteinuria (Figure 6b).

### 2.4. The Effect of AST-120 on Lipid Profile Markers

#### 2.4.1. Serum Total Cholesterol

**Figure 7:**
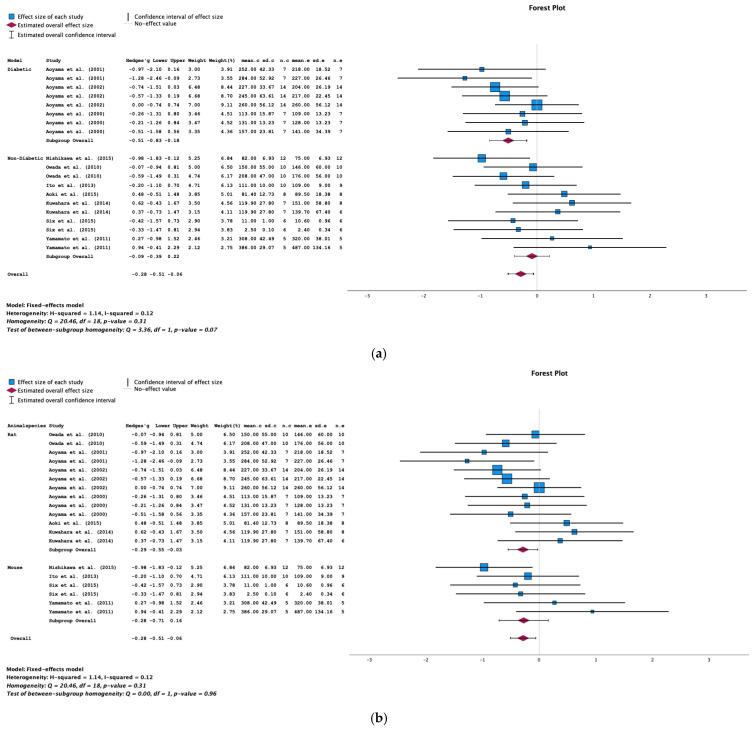

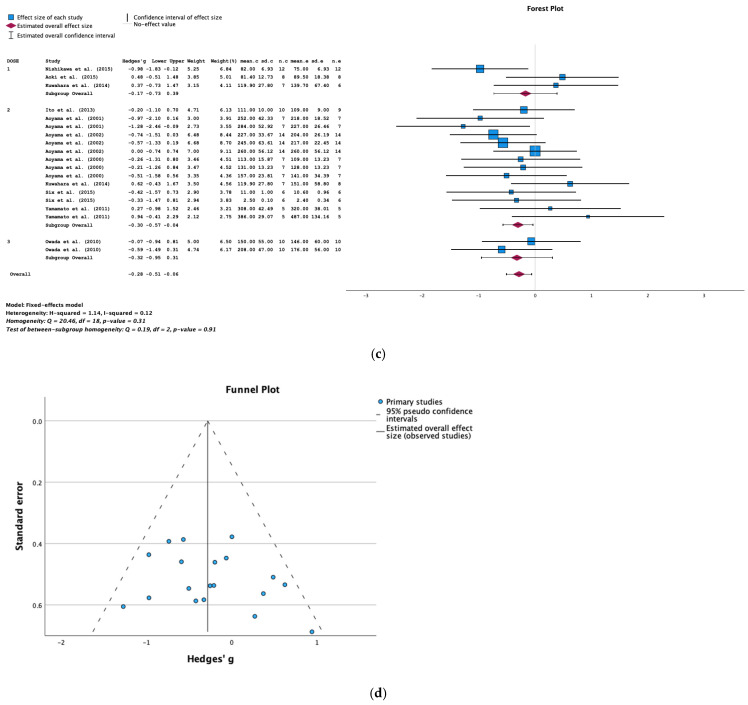
(**a**) A forest plot of data from studies of the effect of AST-120 on the serum total cholesterol levels in animal model subgroups (diabetic vs. nondiabetic animals with CKD) (sd: standart deviation; n: number; c: comparator (CKD group); e: experimental (CKD-AST-120 group)). (**b**) A forest plot of data from studies evaluating the effect of AST-120 on the serum total cholesterol levels in animal species subgroups (rat vs. mouse). (**c**) A forest plot of data from studies evaluating the effect of AST-120 on the serum total cholesterol levels in subgroups of AST-120 doses (1:8% *w*/*w*, 2: 4–5% *w*/*w*, and 3: 4 g/kg BW). (**d**) A funnel plot of studies of the effect of AST-120 on the serum total cholesterol levels in animals with CKD.

Data on the serum total cholesterol level were extracted from 10 studies. The results of the meta-analysis show that AST-120 was associated with a significantly lower serum total cholesterol level in animals with CKD (SMD = −0.28; 95% CI = −0.51, −0.06; *p* = 0.013; I^2^ = 12%) (Figure 7a).

Furthermore, the results of the subgroup analyses showed that the animal model (diabetic CKD; SMD = −0.51; 95% CI = −0.83, −0.18; *p* = 0.002), the species (rats; SMD = −0.29; 95% CI = −0.55, −0.03; *p* = 0.030), and the AST-120 dose (4–5% *w*/*w*; SMD = −0.30; 95% CI = −0.57, −0.04; *p* = 0.025) were influential factors (Figure 7a–c).

The funnel plot and Egger’s test (*p* = 0.114) did not show evidence of publication bias in the studies of the serum total cholesterol level (Figure 7d).

#### 2.4.2. The Serum Triglyceride Level

**Figure 8:**
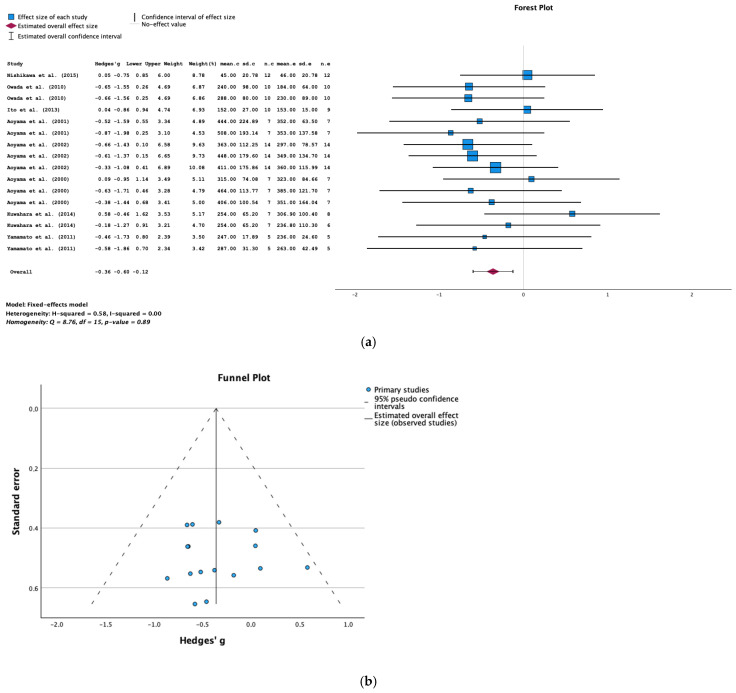
(**a**) A forest plot of data from studies of the effect of AST-120 on the serum triglyceride levels in animals with CKD (sd: standart deviation; n: number; c: comparator (CKD group); e: experimental (CKD-AST-120 group)). (**b**) A funnel plot of studies of the effect of AST-120 on serum triglyceride levels in animals with CKD.

Eight studies provided data on the serum triglyceride level. The results of the meta-analysis show that AST-120 was associated with a significantly lower serum triglyceride level in animals with CKD (SMD = −0.36; 95% CI = −0.60, −0.12; *p* = 0.003; I^2^ = 0%) (Figure 8a).

The funnel plot and Egger’s test (*p* = 0.544) did not show evidence of publication bias in the studies of the serum triglyceride level (Figure 8b).

### 2.5. The Effect of AST-120 on Systolic Blood Pressure

The results of a meta-analysis of data from 13 studies reveal that AST-120 treatment was not significantly associated with the systolic blood pressure in animals with CKD (*p* > 0.05).

## 3. Discussion

The present study is a comprehensive meta-analysis of the efficacy of AST-120 on the renal function markers and lipid profiles in both diabetic and nondiabetic animals with CKD. This meta-analysis extends previous findings on the renoprotective effect of AST-120 treatment in CKD and the considerable improvements in the lipid profile in both diabetic and nondiabetic animals with CKD.

Our results confirm the renoprotective effects of AST-120 treatment in animals with CKD. Subgroup analyses showed that there was no influence of the model’s diabetic status, the AST-120 dose, or the duration of AST-120 treatment. However, the BUN levels were substantially influenced by the treatment duration in animals with CKD. Furthermore, our results indicate that AST-120 treatment is effective in lowering IS levels in both diabetic and nondiabetic animals with CKD.

The evidence gained from the present meta-analysis indicates that AST-120 may be effective in reducing the total cholesterol and triglyceride levels in animals with CKD. Notably, the results of our subgroup analyses show that the diabetic status, animal species (the rat), and dose of AST-120 (4–5% *w*/*w*) influenced the decrement in the total cholesterol level. Several factors (including high plasma cholesterol and triglyceride levels) might contribute to atherosclerotic processes and thus adversely affect cardiovascular function in the presence of renal impairment [7]. It is noteworthy that IS accumulation reportedly prompts foam cell formation by triggering oxidative stress and abnormal lipid metabolism in macrophages [37]. However, it is still not known whether AST-120’s impact on the lipid profile has a positive influence on cardiovascular outcomes. Although the results of some studies show that AST-120 is associated with lower serum lipid levels, the underlying mechanisms have not been fully elucidated [16,21,35]. These positive effects of AST-120 can be attributed to the drug’s efficacy in improving the activities of lipoprotein lipase and hepatic triglyceride lipase, reducing proteinuria, and decreasing VLDL receptor expression [16,38]. Furthermore, our current findings suggest that the beneficial effects of AST-120 on the lipid profile and renal function are independent of a reduction in systolic blood pressure. AST-120’s pleiotropic effects on suppressing oxidative stress and inflammation have been reported previously [39,40].

The potential effects of AST-120 on kidney function and the lipid profile in animals with CKD are clearly due to the reduction in serum IS levels. Indeed, IS induces the aryl hydrocarbon receptor, which is involved in cell differentiation, cell senescence, lipid metabolism, and fibrogenesis [41]. It is also possible that the amelioration of cardiovascular lesions observed during and after treatment with AST-120 also contributes to these beneficial effects [3]. Other mechanisms might be related to AST-120’s effects on the microbiota [42].

Dietary proteins are hydrolyzed by intestinal bacterial enzymes and metabolized into tryptophan, which is then partly converted into indole by resident microbes. This indole passes into the circulation and reaches the liver, where it is metabolized into IS. Similarly, many protein-derived uremic toxins (i.e., p-cresyl sulfate) are metabolized in the gut and then the liver and are excreted by (healthy) kidneys [1,2,3]. Therefore, the accumulation of uremic toxins in the body is closely related to enhanced production and/or reduced excretion. There is growing evidence of a bidirectional relationship between the intestinal microbiota and kidney function. CKD is associated with various alterations in the composition and function of gut microbiota, resulting in intestinal dysbiosis. It has also been reported that intestinal dysbiosis contributes to greater generation of toxic metabolites, including uremic toxins [43]. Concerning indole, a study of 5/6-nephrectomized rats suggested that some Clostridia- and Bacteroidia-affiliated species were associated with indole production in the gut [26]. Moreover, it was found that some microbes involved in the formation of indole (Clostridiaceae, Verrucomicrobiaceae, and Enterobacteriaceae) were abundant in patients with end-stage renal disease [44].

Recent several lines of evidence indicate that AST-120’s ability to counter gut dysbiosis is involved in the beneficial effects seen in renal disease [37,45]. The results of a clinical study showed that the oral administration of AST-120 could induce the production of beneficial metabolites (such as short-chain fatty acids (SCFAs)) by modulating the intestinal microbiota [45]. SCFAs are involved in various metabolic and immunomodulatory functions of importance for renal and intestinal function and integrity [46,47]. Hence, elevated SCFA production is one possible explanation for the beneficial renal and intestinal effects of AST-120.

Although in vivo studies in animals clearly demonstrated the effectiveness of AST-120, these encouraging results have not been confirmed in clinical studies [48,49,50]. It is important to bear in mind that clinical studies had some methodological problems. Moreover, the activation of the aryl hydrocarbon receptor by other tryptophan metabolites (such as kynurenine, which also accumulates in patients with CKD and is associated with CKD complications [51] but is not modulated by AST-120) could explain (at least in part) this discrepancy.

The present meta-analysis has several strengths. We performed a comprehensive meta-analysis of data obtained from 28 preclinical studies in various breeds of rats and mice (with no sex or age restrictions), with various AST-120 doses and treatment durations, and studies in diabetic and nondiabetic animals with CKD. The animals did not receive any concomitant medications, and so the observed effects of the treatment on renal outcomes and lipid markers could be clearly ascribed to AST-120. Our meta-analysis also has several limitations. Firstly, the data were obtained from studies of only two animal models of CKD, which limits the extrapolation of our findings to other animal models of CKD. Secondly, AST-120’s influence on the lipid profile was evaluated by measuring a small set of markers. We expect that future studies will report more laboratory findings of relevance to AST-120’s effects on lipid metabolism and uremic toxins.

## 4. Conclusions

The results of the present meta-analysis confirm the major potential beneficial effects of AST-120 on kidney function and lipid metabolism via a reduction in the serum IS level. These findings will have to be confirmed in subsequent well-designed clinical studies.

## 5. Materials and Methods

### 5.1. Search Strategy

Publications of studies of the impact of AST-120 on animal models of CKD were identified by searching the PubMed and Web of Science databases from inception to 21 August 2024. The following search terms were used: “AST-120” AND “Indoxyl Sulfate” AND “Nephrectomy OR Nephrectomized OR Nephropathy OR Chronic Kidney Disease” AND “Rat OR Mice”.

### 5.2. Study Selection

After the removal of duplicate studies, the titles, abstracts, and full texts of the articles were examined to determine whether the following inclusion criteria were met: (i) the use of an animal model of CKD (rat or mouse with no restrictions on sex, breed, or age) including a remnant kidney after nephrectomy and/or diabetes, (ii) studies where the effects of AST-120 were evaluated, and (iii) studies with at least one of the following outcomes reported: the serum IS level, renal function markers (i.e., serum creatinine, creatinine clearance rate, BUN, and proteinuria), lipid profile markers (i.e., total cholesterol and triglycerides), and systolic blood pressure.

The exclusion criteria were as follows: (i) the lack of an original research article; (ii) the lack of access to the full text; (iii) an unsuitable study type, such as (a) studies with no relevant animal model (i.e., cell culture studies) and (b) studies using agents or chemicals (i.e., streptozotosin, adenine, cyclosporine, and adriamycin) other than AST-120; and (iv) unsuitable study outcomes, the lack of an outcome of interest, or the inability to extract data (Figure 1).

### 5.3. Risk of Bias Assessment of Studies

The risk of bias in each study was rated as low, high, or unclear with regard to each of the following criteria as described in the Systematic Review Centre for Laboratory Animal Experimentation’s risk of bias tool for animal studies: selection bias, performance bias, detection bias, attrition bias, reporting bias, and other bias [52] (Figure S1).

### 5.4. Data Extraction and Conversion

Data were extracted for all outcomes, including the number of animals (n), the mean and the standard deviation (SD), or the standard error of the mean (SEM). The SEM was converted to the SD using the equation SD = SEM×√n.

### 5.5. Statistical Analysis

Meta-analysis was performed using IBM SPSS software version 29 (IBM SPSS Statistics for Windows, IBM, Armonk, NY, USA), and all outcome measures were quoted as continuous variables. All data were processed as the standardized mean difference (SMD) with the 95% confidence interval (CI). Hedge’s g was used to calculate the effect size for each study. A funnel plot of Hedge’s g against the standard error was used to assess visually whether publication bias was likely. Possible publication bias was also analyzed using Egger’s regression-based test; a *p* value > 0.05 indicated that there was no publication bias. Interstudy heterogeneity was assessed by calculating the I^2^ homogeneity statistic. If I^2^ was greater than 50%, the random effects model was used; if not, the fixed effects model was implemented. To explain interstudy heterogeneity and to examine the influence of certain variables (including the AST-120 dose, the animal species (rats vs. mice), the diabetic status of the animal model (diabetic CKD vs. nondiabetic CKD), and the duration of the AST-120 treatment, subgroup meta-regression analyses were performed. The threshold for statistical significance was set to *p* < 0.05. All tests were two-sided.

## Figures and Tables

**Table 1:** The characteristics of the studies included in the meta-analysis.

Study (Year Published)	Species (Sex)	Animal Model (Diabetic or Nondiabetic CKD)	Numbers of Animals in Comparator (CKD) Group and Experimental (CKD-AST-120) Group	AST-120 Dose and Duration(s) of Treatment (Weeks)	Outcome Markers
Miyazaki et al. (2000) [6]	SD rats (male)	5/6 nephrectomy (nondiabetic CKD)	CKD group (n = 5) CKD-AST-120 group (n = 5)	0.4 g/100 g BW/day for 2, 6, or 10 weeks	1, 2, 3, 4, 5
Lekawanvijit et al. (2012) [10]	SD rats (male)	5/6 nephrectomy (nondiabetic CKD)	CKD group (n = 17) CKD-AST-120 group (n = 13)	chow containing 8% *w*/*w* AST-120 for 8 or 12 weeks	1, 2, 3, 5, 8
Bolati et al. (2012) [11]	SD rats (male)	5/6 nephrectomy (nondiabetic CKD)	CKD group (n = 8) CKD-AST-120 group (n = 8)	4 g/kg/day for 10 weeks	1, 2, 3
Tumur et al. (2008) [12]	SD rats (male)	4/5 nephrectomy (nondiabetic CKD)	CKD group (n = 6) CKD-AST-120 group (n = 6)	4 g/kg/day for 16 weeks	1, 2, 3, 4, 5
Komiya et al. (2004) [13]	SD rats (male)	3/4 nephrectomy (nondiabetic CKD)	CKD group (n = 11) CKD-AST-120 group(n = 11)	4 g/kg for 18 weeks	1, 2, 4, 5, 8
Kobayashi et al. (2002) [14]	SD rats (male)	3/4 nephrectomy (nondiabetic CKD)	CKD group (n = 9) CKD-AST-120 group (n = 9)	0.4 g/100 g BW for 19 weeks	1, 4, 8
Nakagawa et al. (2006) [15]	SD rats (male)	5/6 nephrectomy (nondiabetic CKD)	CKD group (n = 7) CKD-AST-120 group (n = 7)	diet containing 5% AST-120 for 6, 12, or 18 weeks	1, 2, 3, 4, 5
Aoyama et al. (2001) [16]	OLETF rats (male)	Unilateral nephrectomy OLETF (1/2NxOLETF) rats (diabetic CKD)	CKD group (n = 7) CKD-AST-120 group (n = 7)	diet containing 5% AST-120 20 weeks, 36 weeks	1, 2, 3, 4, 5, 6, 7
Aoyama et al. (2002) [17]	OLETF rats (male)	Unilateral nephrectomy OLETF (1/2NxOLETF) rats (diabetic CKD)	CKD group (n = 14) CKD-AST-120 group (n = 14)	chow containing 5% AST-120 for 16, 32, or 48 weeks	1, 2, 3, 4, 5, 6, 7
Aoyama et al. (2000) [18]	OLETF rats (male)	OLETF rats (diabetic CKD)	CKD group (n = 7) CKD-AST-120 group (n = 7)	chow containing 5% AST-120 for 25, 45, or 65 weeks	1, 2, 3, 4, 5, 6, 7
Anraku et al. (2017) [19]	Rats (male)	5/6 nephrectomy (nondiabetic CKD)	CKD group (n = 5) CKD-AST-120 group (n = 5)	40 mg/kg BW for 4 weeks	1, 2, 4
Shimoishi et al. (2007) [20]	Wistar rats (male)	5/6 nephrectomy (nondiabetic CKD)	CKD group (n = 7) CKD-AST-120 group (n = 7)	4.5 g/kg BW for 10 weeks	1, 2, 3, 4, 5
Owada et al. (2010) [21]	SD rats (male)	5/6 nephrectomy (nondiabetic CKD)	CKD group (n = 10) CKD-AST-120 group (n = 10)	4 g/kg BW for 10 or 20 weeks	2, 6, 7
Okada et al. (2005) [22]	SD rats (male)	2/3 nephrectomy (2/3 Nx) (nondiabetic CKD) 4/5 nephrectomy (4/5 Nx)(nondiabetic CKD)	CKD group (n = 15) CKD-AST-120 group (n = 15) CKD group (n = 15) CKD-AST-120 group (n = 15)	1 g/day for 8 weeks	2, 3, 4, 5
Namikoshi et al. (2009) [23]	Wistar rats (male)	Subtotal nephrectomy (nondiabetic CKD)	CKD group (n = 12) CKD-AST-120 group (n = 12)	0.4 g/100 g BW for 2 weeks	2, 3, 4, 5, 8
Yamazaki et al. (2014) [24]	SD rats (male)	3/4 nephrectomy (nondiabetic CKD)	CKD group (n = 9) CKD-AST-120 group (n = 9)	0.4 g/100 g BW for 10 weeks	2, 4, 8
Kikuchi et al. (2010) [25]	SD rats (male)	5/6 nephrectomy (nondiabetic CKD)	CKD group (n = 16) CKD-AST-120 group (n = 16)	chow containing 5% AST-120 for 3 days (0.4 weeks)	1
Kikuchi et al. (2017) [26]	SD rats (male)	5/6 nephrectomy (nondiabetic CKD)	CKD group (n = 7) CKD-AST-120 group (n = 6)	chow containing 8% *w*/*w* AST-120 for 24 weeks	2, 3, 5
Ito et al. (2013) [27]	C57BL/6J mice (male)	Right nephrectomy (nondiabetic CKD)	CKD group (n = 10) CKD-AST-120 group (n = 9)	diet containing 5% (*w*/*w*) AST-120 for 4 weeks	1, 6, 7, 8
Nishikawa et al. (2015) [28]	C57BL/6J mice (male)	Subtotal nephrectomy (nondiabetic CKD)	CKD group (n = 12) CKD-AST-120 group (n = 12)	diet containing 8% *w*/*w* AST-120 for 20 weeks	4, 6, 7, 8
Aoki et al. (2015) [29]	SD rats (male)	5/6 nephrectomy (nondiabetic CKD)	CKD group (n = 8) CKD-AST-120 group (n = 8)	chow containing 8% *w*/*w* AST-120 for 4 weeks	2, 3, 4, 6, 8
Enoki et al. (2023) [30]	C57BL/6JJmsSIc Mice (male)	5/6 nephrectomy (nondiabetic CKD)	CKD group (n = 8) CKD-AST-120 group (n = 7)	chow containing 8% *w*/*w* AST-120 for 4 weeks	1, 2, 4
Kuwahara et al. (2014) [31]	SD rats (male)	4/5 nephrectomy (nondiabetic CKD)	CKD group (n = 7) CKD-AST-120 (4% ) group (n = 8) CKD-AST-120 (8%) group (n = 6)	diet containing 4% and 8% AST-120 for 8 weeks	1, 2, 3, 4, 5, 6, 7, 8
Lin et al. (2020) [32]	SD rats (male)	5/6 nephrectomy (nondiabetic CKD)	CKD group (n = 5) CKD-AST-120 group (n = 4)	chow containing 8% *w*/*w* AST-120 for 12 weeks	1, 2, 4, 8
Taki and Niwa (2007) [33]	SD rats (male)	4/5 nephrectomy (nondiabetic CKD)	CKD group (n = 6) CKD-AST-120 group (n = 6)	4 g/kg for 8 or 16 weeks	4, 5
Yamamato et al. (2011) [34]	Apolipoprotein E- knock-out mice (female)	-Unilateral nephrectomy (nondiabetic CKD) -Subtotal nephrectomy (nondiabetic CKD)	CKD group (n = 5) CKD-AST-120 group (n = 5) CKD group (n = 5) CKD-AST-120 group (n = 5)	5% (*w*/*w*) for 17 weeks	3, 6, 7, 8
Six et al. (2015) [35]	Apolipo-protein E knock-out mice (female) C57BL/6J mice (female)	Left total nephrectomy (nondiabetic CKD)	-CKD group (n = 6) CKD-AST-120 group (n = 6) -CKD group (n = 6) CKD-AST-120 group (n = 6)	4% *w*/*w* for 8 weeks	1, 6, 8
Nakada et al. (2019) [36]	ApoE-deficient mice (male)	5/6 nephrectomy (nondiabetic CKD)	CKD group (n = 8) CKD-AST-120 group (n = 8)	chow containing 8% *w*/*w* AST-120 for 12 weeks	1, 4, 8

1: serum IS, 2: serum creatinine, 3: creatinine clearance rate, 4: BUN, 5: proteinuria, 6: serum total cholesterol, 7: serum triglyceride, 8: systolic blood pressure, BW: body weight, SD: Sprague Dawley, OLETF: Otsuka Long-Evans Tokushima Fatty.

## Data Availability

The original contributions presented in this study are included in the article/supplementary material. Further inquiries can be directed to the corresponding author.

## Supplementary Materials

The following supporting information can be downloaded at https://www.mdpi.com/article/10.3390/toxins16120544/s1, Figure S1: A risk of bias summary for the included studies; Table S1: The Preferred Reporting Items for Systematic Review and Meta-Analyses (PRISMA) checklist.
